# Enhanced Anti-Lung Cancer Efficacy of Neo-BCV Combined with Cisplatin: Immune Activation and Tumor Microenvironment Remodeling

**DOI:** 10.3390/vaccines14050436

**Published:** 2026-05-13

**Authors:** Quexun Cai, Qianli Yang, Kangrui Zhang, Zhengyue Fei, Ruochen Zhao, Tao Lu, Kecheng Xu, Zhenyi Wang, Peihua Lu

**Affiliations:** 1 School of Medicine, Jiangnan University, Wuxi 214122, China; 18760463984@139.com; 2 The Affiliated Wuxi People’s Hospital of Nanjing Medical University, Wuxi People’s Hospital, Wuxi Medical Center, Nanjing Medical University, Wuxi 214023, China; qianliyang0605@163.com (Q.Y.); zkr0301@163.com (K.Z.); m19822650170@163.com (Z.F.); dfmy1874@gmail.com (R.Z.); 3 School of Life Science, Beijing University of Chinese Medicine, Beijing 100029, China; taolu@bucm.edu.cn; 4 Oncology Department, National Key Clinical Specialty (Oncology), Biomedical Translational Research Institute, Fuda Cancer Hospital, Jinan University, Guangzhou 510632, China; xukc@vip.163.com; 5 National Research Centre for Translational Medicine at Shanghai, State Key Laboratory of Medical Genomics, Research Unit of Hematologic Malignancies Genomics and Translational Research of Chinese Academy of Medical Sciences, Shanghai Institute of Hematology, Ruijin Hospital, Shanghai Jiao Tong University School of Medicine, Shanghai 200025, China; 13661734438@163.com

**Keywords:** vaccine, cisplatin, lung cancer, immunology, tumor microenvironment

## Abstract

Background: Lung cancer is the top cause of cancer-related mortality globally, and chemo-immunotherapy is a core therapeutic strategy for it. The novel bacterial composite vaccine (Neo-BCV) we developed previously can activate anti-tumor immunity. This study explored its synergistic anti-tumor effect with cisplatin (CDDP), along with the underlying immunomodulatory mechanisms and molecular regulatory networks. Methods: A murine Lewis lung cancer (LLC) model was established to evaluate the efficacy of the combination therapy. Flow cytometry and multiplex cytokine assay were used to detect immune cell subsets and functional molecules in the spleen, serum and tumor tissues. RNA-sequencing (RNA-seq) was used to elucidate the molecular regulatory networks following the combination therapy in the tumor tissues. Body weight, blood indexes, serum biochemistry and H&E staining were monitored to verify biosafety. Results: Neo-BCV combined with CDDP achieved an 87.77% tumor growth inhibition rate, showing the most significant anti-tumor effect. The combination promoted DC maturation, enhanced effector immune cell infiltration, reduced immunosuppressive cells, upregulated Th1-type cytokines and downregulated CD8^+^ T cell surface PD-1. RNA-seq confirmed enrichment of multiple immune effector pathways, supporting tumor immune microenvironment remodeling. The combination alleviated CDDP-induced weight loss, had no obvious adverse effects on physiological indicators, and exhibited good biosafety. Conclusions: Neo-BCV combined with CDDP achieves enhanced anti-tumor efficacy and favorable biosafety in murine lung cancer models by regulating immune cell subsets and activating immune-related molecular pathways, providing a solid preclinical basis for its clinical translation in lung cancer treatment.

## 1. Introduction

Lung cancer is a malignant tumor with one of the highest incidences and mortality rates worldwide, posing a severe threat to human health and life security. Among them, non-small cell lung cancer (NSCLC) accounts for more than 85% of all lung cancer cases. Due to its insidious early symptoms, high malignancy, and strong invasive and metastatic potential, most patients are diagnosed at advanced stages and thus miss the opportunity for radical surgical treatment [1,2,3]. Traditional clinical therapeutic approaches include surgical resection, radiotherapy and chemotherapy, yet all have obvious limitations. Even immune checkpoint inhibitors, which have achieved remarkable clinical progress, often exhibit relatively limited efficacy in the treatment of advanced lung cancer due to issues such as tumor heterogeneity and drug resistance, highlighting an urgent need for the development of novel and effective therapeutic strategies [4,5,6].

Platinum-based chemotherapeutic drugs represented by cisplatin (CDDP) serve as the first-line foundational regimen for the treatment of advanced lung cancer. Despite their extensive clinical application, their use is restricted by multiple factors. Their core mechanism of action is to form cross-links with purine bases on tumor cell DNA, interfere with DNA replication and repair processes, and ultimately induce programmed apoptosis of tumor cells [7,8]. In addition, CDDP can induce anti-tumor immunomodulation through mechanisms such as upregulating the expression of MHC class I molecules, recruiting effector cells, enhancing the lytic activity of cytotoxic effectors, and releasing damage-associated molecular patterns (DAMPs) [9,10]. However, the clinical application of high-dose CDDP is severely limited by multiple problems: first, it easily induces significant adverse reactions such as gastrointestinal toxicity, nephrotoxicity and myelosuppression [11,12,13,14]; second, long-term use tends to lead to tumor drug resistance and attenuate chemotherapeutic effects [15,16]. These limitations further highlight the necessity of rational optimization of CDDP dosage and the development of novel therapeutic strategies.

In recent years, the rise of tumor immunotherapy has brought a breakthrough revolution to lung cancer treatment. Accumulating evidence has indicated that the combination of immunotherapy and traditional chemotherapeutic drugs can achieve better therapeutic outcomes for cancer [17,18,19]. Although programmed cell death protein 1 (PD-1) inhibitors combined with chemotherapy yield a relatively high objective tumor response rate, they suffer from shortened duration of efficacy, along with various immune-related adverse events (irAEs), toxicity superposition and high costs [20,21,22]. Against this background, therapeutic tumor vaccines have emerged as an important direction to break through the existing therapeutic bottleneck by virtue of their unique advantage of inducing durable systemic anti-tumor immunity [23,24,25]. The application of tumor vaccines can be traced back to the “Coley’s toxins” developed by William Coley a century ago, which successfully treated advanced cancer by activating immune responses with inactivated bacteria and laid the foundation for bacterial immunotherapy [26]. Subsequently, Bacillus Calmette-Guérin (BCG) was proven to enhance anti-tumor immunity by activating bone marrow hematopoietic stem cells and progenitor cells, and its standardized application in bladder cancer treatment further confirmed that bacterial preparations can exert anti-tumor effects by remodeling the tumor microenvironment [27,28,29]. Compared with single bacterial preparations, bacterial composite vaccines integrate a variety of immune activation components and can enhance efficacy and synergistic potential through different mechanisms. In addition, the addition of β-glucan can bind to receptors on the surface of immune cells, activate the pro-inflammatory response of innate immune cells, and further enhance the host’s anti-tumor defense capacity [30,31].

This study focused on the novel bacterial composite vaccine (Neo-BCV) we previously developed, which is mainly composed of heat-inactivated strains of *diphtheria*, *pertussis*, *tetanus*, *typhoid-paratyphoid*, and *Staphylococcus aureus* as well as a glucan adjuvant [32]. Preliminary studies have found that Neo-BCV can activate and induce the differentiation and maturation of dendritic cells, thereby triggering anti-tumor immune responses [33]. In addition to directly killing tumor cells, CDDP also exerts anti-tumor immunomodulatory effects [8,9], which provides a theoretical basis for the synergistic anti-tumor effect of the combination of the two agents. However, under the current research background, the specific synergistic anti-tumor effect, cellular mechanism of immunomodulation, and molecular regulatory network supporting this process of the combined application of Neo-BCV and CDDP remain unclear. Therefore, this study aimed to systematically evaluate the anti-tumor effect of this combination therapy in a lung cancer model, clarify its remodeling characteristics of the tumor immune microenvironment from both cellular phenotypic and molecular mechanistic perspectives by combining flow cytometry and RNA-sequencing (RNA-seq), and comprehensively assess its in vivo biosafety. This research provides a more comprehensive theoretical basis and experimental reference for the combination therapy strategy of the two drugs.

## 2. Materials and Methods

### 2.1. Experimental Cell Lines, Animals and Ethics

The Lewis lung cancer (LLC) cell line (Crondabio, Shanghai, China) was authenticated by short tandem repeat (STR) analysis to ensure no cross-contamination. Derived from spontaneous lung adenocarcinoma of C57BL/6 mice, this cell line was cultured in complete DMEM (90% DMEM high-glucose medium + 10% FBS + 1% double antibody) and is widely used in lung cancer-related research.

Specific pathogen-free (SPF) healthy male/female C57BL/6 mice, aged 6–8 weeks with a body weight of approximately 20~25 g, were purchased from Changzhou Cavens Laboratory Animal Co., Ltd. (Changzhou, China). (Production License No.: SCXK (Su) 2021-0013). All experimental mice were raised in the Animal Experiment Center of Wuxi People’s Hospital in accordance with SPF grade standards, with 5–6 mice per cage. The animal room was maintained with a 12 h light/dark cycle, a temperature of 25 °C and a relative humidity of 50~70%. Mice were fed with standard chow and provided with sterile drinking water ad libitum. Experiments were initiated one week after the mice were housed. Before all surgical procedures, mice were anesthetized with isoflurane in an induction chamber, and cervical dislocation was performed for euthanasia when the mice lost all sensation after the operation. All experimental animal research protocols were reviewed and approved by the Scientific Research Ethics Committee of Wuxi People’s Hospital (Ethics Approval No.: DL2025024).

### 2.2. Preparation of Neo-BCV and CDDP

Neo-BCV is a complex bacterial suspension formulation consisting primarily of six heat-inactivated bacterial components: (1) *Bordetella pertussis* at a concentration of 9 × 10^9^ CFU/mL; (2) *Corynebacterium diphtheriae* endotoxin at 20 Lf/mL; (3) *Clostridium tetani* endotoxin at 5 Lf/mL; (4) *Salmonella typhi* at 3 × 10^8^ CFU/mL; (5) *Salmonella paratyphi* types A and B, each at 1.5 × 10^8^ CFU/mL; and (6) 10% *Staphylococcus aureus* solution at 1 × 10^9^ CFU/mL, supplemented with 0.2 g/mL glucan as an adjuvant. All bacterial strains and related preparations were obtained from the National Institutes for Food and Drug Control (Silver Spring, MD, USA). Technical support and bacterial culture services were provided by Zhejiang Weixin Biological Pharmaceutical Co., Ltd. (Hangzhou, China). The prepared vaccine was confirmed to pose no infection risk.

Preparation of CDDP: CDDP (Solarbio, Beijing, China) was dissolved in N, N-dimethylformamide (DMF) (Macklin Biochemical Technology Co., Ltd., Shanghai, China) to prepare a CDDP stock solution with a concentration of 10 mg/mL. Immediately before injection, the above stock solution was secondarily diluted with sterile normal saline according to the doses required by the experimental design.

### 2.3. Establishment of Animal Model and Grouping and Administration

LLC cells in the logarithmic growth phase were digested with 0.25% Trypsin-EDTA (Thermo Fisher, Waltham, MA, USA), resuspended in PBS and adjusted to a cell concentration of 1 × 10^6^ cells/mL. A 100 μL single-cell suspension was subcutaneously injected into the right axilla of mice (1 cm below the axilla) to establish the subcutaneous xenograft tumor model for all mice.

When tumors became palpable (about 4–6 days, tumor volume reached 60–70 mm^3^ measured by a vernier caliper), the mice were randomly divided into 4 groups (*n* = 8 per group): (1) Control group: 0.1 mL normal saline per mouse every 3 days (intraperitoneal injection), 4 times in total, (2) Neo-BCV group: 0.1 mL Neo-BCV per mouse every 3 days (subcutaneous injection), 4 times in total, (3) CDDP group: based on previous studies, 3 mg CDDP/kg per mouse every 3 days (intraperitoneal injection) [34], 4 times in total, (4) Neo-BCV combined with CDDP group (abbreviated as BCV-CDDP group): the administration route and dosage were the same as the Neo-BCV group and CDDP group. Based on previous studies and our experimental optimization, CDDP was administered first, with a 24-h interval between the two drugs [35,36]. The body weight of mice was dynamically monitored daily using an electronic analytical balance.

For ethical considerations, the terminal body weight was recorded when the tumor volume reached 1000 mm^3^, mice developed cachexia, showed no response to noxious stimuli, lost more than 20% of their body weight, or the tumor weight exceeded 10% of the normal body weight. The wet weight of the liver, kidney and spleen was accurately weighed to calculate the organ index: (organ weight/terminal body weight × 100%). Subcutaneous tumors and related tissues including heart, liver, spleen, kidney and lung were dissected and collected. Tumor images were taken, and tumor weight was measured to calculate the tumor inhibition rate: [(average tumor weight of the control group − average tumor weight of the treatment group)/average tumor weight of the control group] × 100%. Tissue samples were frozen at −80 °C or fixed with 4% paraformaldehyde for subsequent experimental analysis.

### 2.4. H&E Staining

The fixed tissue samples were thoroughly washed under a continuous stream of water to remove any lingering fixative. This was followed by dehydration in an ascending series of ethanol, paraffin infiltration, and subsequent microtomy. The resulting sections underwent deparaffinization in xylene and were rehydrated through a descending alcohol gradient prior to hematoxylin staining. After a brief rinse, eosin was applied as a counterstain. Finally, the slides were dehydrated, cleared in xylene, and permanently sealed with neutral balsam. Histopathological assessment was performed using light microscopy, with typical micrographs captured for further analysis.

### 2.5. TUNEL Staining

Paraffin sections were deparaffinized and rehydrated. Proteinase K working solution was added for antigen retrieval, and 0.1% Triton X-100 (Thermo Fisher, Waltham, MA, USA) permeabilization working solution was added for cell permeabilization, followed by adding buffer to cover the tissue and equilibrating at room temperature. According to the number of sections and tissue size, an appropriate amount of reaction solution from the TUNEL kit (KeyGEN Biotech, Nanjing, China) was added, and DAPI stain (Erwan Biotechnology Co., Ltd., Shanghai, China) was dripped for nuclear counterstaining. After mounting, the sections were observed under a fluorescence microscope (OLYMPUS BX53, Olympus, Tokyo, Japan) and images were captured for result interpretation.

### 2.6. Immunohistochemistry

Tissues were fixed with 4% paraformaldehyde for 24 h, then embedded in paraffin and sectioned. After deparaffinization and rehydration of paraffin sections, heat-induced antigen retrieval was performed to expose target proteins. After natural cooling, sections were incubated in 3% hydrogen peroxide solution at room temperature in the dark to block endogenous peroxidase activity. After blocking with blocking solution (rabbit serum for goat-derived primary antibodies, BSA for other sources), rabbit anti-Ki67 monoclonal antibody (1:200, Solarbio, Beijing, China) overnight at 4 °C. Subsequently, the sections were incubated with HRP-conjugated goat anti-rabbit IgG antibody (1:500, Solarbio, Beijing, China) for 1 h at room temperature. Finally, the signal was visualized using a DAB substrate kit (Solarbio, Beijing, China). Hematoxylin was then used for nuclear counterstaining, and after dehydration and mounting, the sections were observed under a microscope, and images were captured using a Nikon DS-Ri2 digital microscope camera system (Nikon, Tokyo, Japan).

### 2.7. Mouse Complete Blood Count and Plasma Sample Detection

At the end of the experiment, orbital venous plexus puncture was performed to collect blood from anesthetized C57BL/6 mice, and cervical dislocation was immediately performed for euthanasia after blood collection. Whole blood samples were allowed to stand at room temperature for 15–30 min and then thoroughly mixed, and detected using a veterinary automatic hematology analyzer (Mindray Animal, Shenzhen, China), with results reviewed by blood smear microscopy. Plasma was collected using the same puncture method, allowed to stand at room temperature for natural coagulation and then centrifuged. The upper pale yellow clear plasma was aspirated, aliquoted, and detected according to the instructions of the biochemical kits (Jiancheng Bioengineering Institute, Nanjing, China), with corresponding standard curves established for quantitative calculation. Extrusion of the eyeball and violent shaking were avoided throughout the process to prevent hemolysis from affecting the detection results.

### 2.8. Flow Cytometry of Mouse Tumor and Spleen Tissues

Preparation of digestion solution: Collagenase (Type I, II and IV) was thoroughly mixed with Gibco™ RPMI 1640 (Thermo Fisher, Waltham, MA, USA), and DNase I (STEMCELL Technologies, Vancouver, BC, Canada) was added to a final concentration of 1 μg/mL. Tumor tissues were digested with shaking in the above digestion solution for 1.5 h (37 °C), and filtered through a 200-mesh sieve to prepare single-cell suspensions. Spleen tissues were ground on a 100-mesh cell sieve, red blood cells were removed from the collected filtrate with red blood cell lysis buffer to prepare single-cell suspensions, and the cell concentration was adjusted to 1 × 10^7^ cells/mL. Four mixed antibody solutions were then prepared: Mixed Solution 1 (CD45-APC-CY7, LY6C-AF700, LY6G-Percp-CY5.5, F4-80-APC, CD335-BV650, CD11b-PE-dazzle594, CD3-FITC, CD8a-BV605, CD4-PE-CY7, PD-1-BV421, FOXP3-PE); Mixed Solution 2 (CD45-PerCP-CY5.5, B220-BV786, CD23-BV421, CD5-AF700, CD19-APC-CY7, IgM-PE-CY7, IgD-FITC, CD21/CD35-PE, CD1d-APC); Mixed Solution 3 (CD45-APC-CY7, CD11b-BV421, CD3-FITC, CD4-PE-CY7, CD8a-BV605, TNF-α-PE-dazzle594, IFN-γ-PerCP-CY5.5, GRAMZYME B-APC); Mixed Solution 4 (CD45-APC-CY7, CD11c-AF700, CD80-PE-CY7, CD86-PE). All the antibodies were from BioLegend, San Diego, CA, USA. Among them, Mixed Solutions 1, 2 and 3 were used for flow cytometry of tumor tissues: Mixed Solution 1 for surface and intracellular Foxp3 staining, Mixed Solution 2 for surface staining, and Mixed Solution 3 for T cell activation and intracellular cytokine staining. Mixed Solution 4 was used for flow cytometry of spleen tissues.

After preparation of the mixed solutions, 100 μL of single-cell suspension was taken and incubated with the corresponding flow cytometry antibody panel at 4 °C in the dark for 30 min (fixation and permeabilization were required for intracellular factor detection). After washing with PBS, the samples were detected on the flow cytometer (Beckman Coulter CytoFlex (Indianapolis, IN, USA), 3-laser and 13-channel). FlowJo v10.8.1 was used to analyze the maturation of splenic DCs (CD11c^+^CD80^+^CD86^+^), the proportion of tumor-infiltrating immune cell subsets (CD4^+^/CD8^+^ T cells, NK cells, M1/M2-type macrophages, B cells, regulatory T cells, myeloid-derived suppressor cells) and the expression levels of functional molecules (PD-1, TNF-α, IFN-γ, Granzyme B).

### 2.9. Flow Cytometric Multiplex Cytokine Detection of Mouse Serum

Measurement of mouse serum cytokines (TNF-α, IFN-γ, IFN-β, IL17A) was performed using magEasyQPlex Mouse 10-plex Flow Assay Kit (CLEM010, Laizee Biotech, Shanghai, China) on Attune NxT Flow Cytometer. Briefly, 25 μL serum samples were incubated for 2 h with magnetic mixture bead sets which were uniquely color coded with fluorescent dyes and pre-coated with analyte-specific antibodies. After magnetic washing, biotinylated detection antibodies and PE-streptavidin were sequentially added. The Attune NxT’s red laser identified bead populations while the blue laser quantified PE fluorescence, with concentrations calculated from standard curves.

### 2.10. RNA Extraction and Sequencing of Tumor Tissues

Total RNA was extracted from tumor tissues using TRIzol^®^ reagent (Invitrogen, Waltham, MA, USA) following the manufacturer’s protocol. The extracted RNA was treated with DNase I (STEMCELL Technologies, BC, Canada) to eliminate genomic DNA contamination. RNA integrity and concentration were determined using an Agilent 2100 Bioanalyzer (Agilent, Santa Clara, CA, USA) and a NanoDrop ND-2000 Spectrophotometer (NanoDrop Technologies, Wilmington, DE, USA), respectively. High-quality RNA samples (OD260/280 = 1.8–2.2, OD260/230 ≥ 2.0, RIN ≥ 6.5, 28S:18S ≥ 1.0, total RNA amount > 10 μg) were selected for sequencing library construction. Transcriptome sequencing libraries were built with 1 μg of total RNA per sample using the Illumina TruSeq™ RNA Sample Preparation Kit (Illumina, San Diego, CA, USA). After quantification by a TBS380 Fluorometer, paired-end sequencing (150 bp × 2) was conducted on the Illumina platform by Biochip Co., Ltd. (Shanghai, China).

After sequencing, raw data were quality-controlled and filtered using Trimmomatic software version 0.39 (adapter sequences and low-quality reads removed). Clean reads were aligned to the mouse reference genome (mm10) via Tophat software version 2.1.1, and transcripts were assembled using Cufflinks software version 2.2.1. Differentially expressed genes (DEGs) were filtered by edgeR software version R3.6.3 with the criteria: |log2FC| ≥ 1, FDR < 0.05. To explore the biological functions of DEGs, Gene Ontology (GO) enrichment analysis and Kyoto Encyclopedia of Genes and Genomes (KEGG) pathway analysis were conducted using Goatools (https://github.com/tanghaibao/Goatools) (accessed on 28 September 2025) and KOBAS (http://bioinfo.org/kobas3/), (accessed on 28 September 2025) respectively.

### 2.11. Statistical Analysis

All experimental results were expressed as mean ± standard deviation (Mean ± SD), and statistical analyses were performed using GraphPad Prism 10.0 software. One-way analysis of variance (one-way ANOVA) was used for comparisons among multiple groups and survival curves were analyzed by the Log-rank test for trend. A *p*-value < 0.05 was considered statistically significant. For each group, *n* denotes the number of biologically independent mice, with the number of biological replicates indicated in the figure legend. All experiments were independently repeated 3 times for stability, and representative results are shown in the figures.

## 3. Results

### 3.1. Synergistic Anti-Tumor Efficacy of Neo-BCV Combined with CDDP Therapy

**Figure 1:**
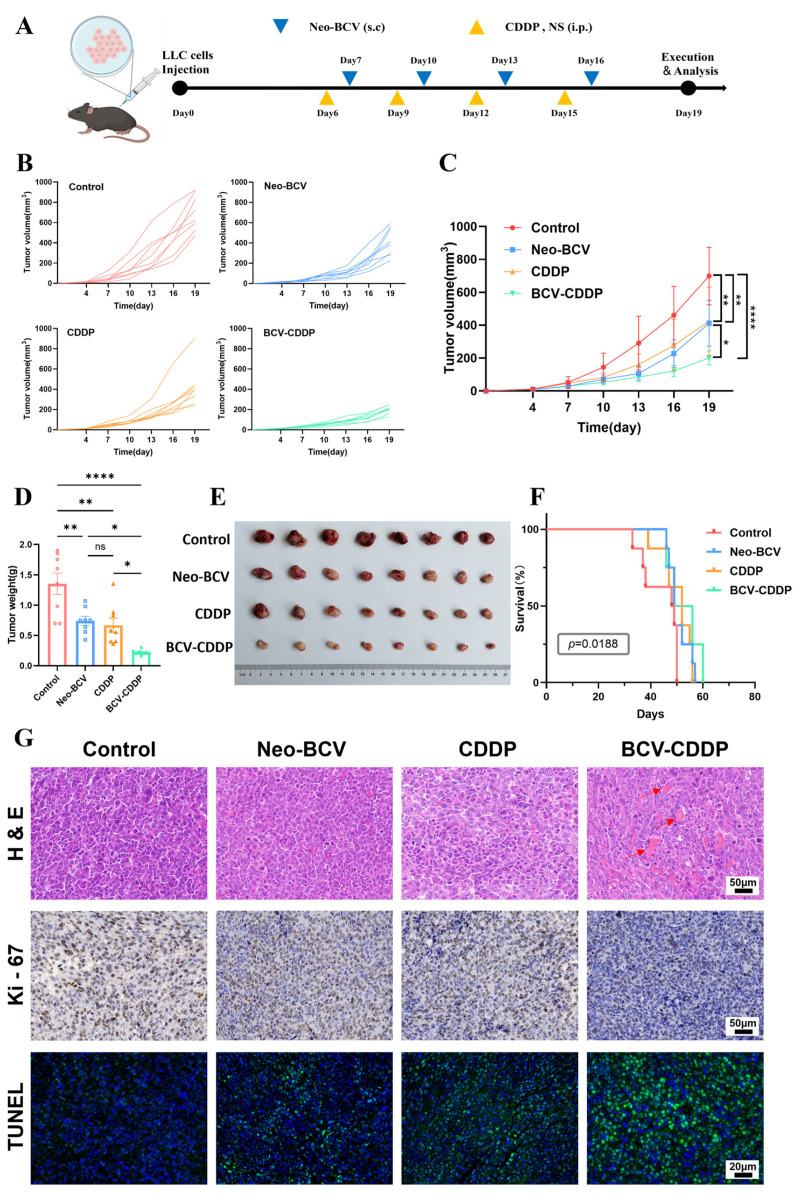
Inhibitory effect of BCV-CDDP on tumor growth. (**A**) Experimental protocol of the Lewis tumor-bearing mouse model. (**B**) Dynamic changes in tumor volume of mice in each group during treatment (*n* = 8). (**C**) Comparison of average tumor volume of mice in each group (*n* = 8). (**D**) Quantitative comparison of average tumor weight in each group at the end of treatment (*n* = 8). (**E**) Gross images of tumor tissues in each group (*n* = 8). (**F**) Comparison of mouse survival curves among different groups (*n* = 8). (**G**) Representative images of H&E, Ki-67 and TUNEL staining of tumor tissues in each group (*n* = 4). Data are presented as mean ± SD. A *p*-value < 0.05 was considered statistically significant, with the significance levels marked as * *p* < 0.05, ** *p* < 0.01, **** *p* < 0.0001, and ns means no significance.

To evaluate the in vivo synergistic anti-tumor potential of Neo-BCV combined with CDDP, a subcutaneous xenograft tumor model of LLC in C57BL/6 mice was established in this study. Mice were randomly divided into four groups for drug treatment (Figure 1A), and tumor growth curves were monitored (Figure 1B). Results at the end of treatment showed that compared with the control group, the tumor growth rate in the BCV-CDDP group was significantly slowed, with a tumor growth inhibition rate of up to 87.77%, which was significantly higher than that in the Neo-BCV group (66.07%) and the CDDP group (62.18%). In addition, the tumor volume and weight in the BCV-CDDP group were the smallest among all the groups with statistically significant differences (Figure 1C–E). Corresponding to the highest tumor inhibition rate and smallest tumor volume and weight, the BCV-CDDP group also exhibited the longest survival time (Figure 1F).

Subsequently, histological analysis of tumor samples was performed to further evaluate the anti-tumor effect (Figure 1G). H&E staining results showed that extensive necrotic areas appeared in the tumor tissues of the BCV-CDDP group with a reduction in viable tumor cells, and Ki-67 immunohistochemical staining revealed the most prominent downregulation in this group. Furthermore, TUNEL assay showed the strongest green fluorescent signal intensity and the largest number of apoptotic cells in the BCV-CDDP group. In summary, these results indicated that the combination therapy exerted the most significant inhibitory effect on tumor cell proliferation.

### 3.2. Effects of Neo-BCV Combined with CDDP Therapy on Systemic Immunity

**Figure 2:**
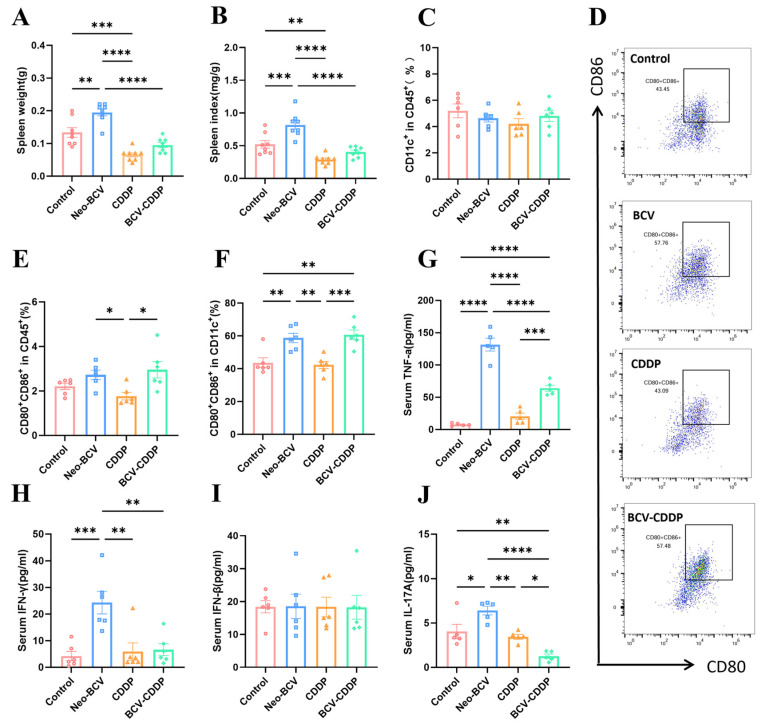
Comparison of splenic and serum immune function in each group. (**A**) Spleen weight of mice in each group (*n* = 8). (**B**) Spleen index of mice in each group (*n* = 8). (**C**) Quantification of CD45^+^ CD11c^+^ cells in the spleen (*n* = 6). (**D**) Proportion of CD11c^+^CD80^+^CD86^+^ cells in the spleen (*n* = 6). (**E**) Quantification of CD45^+^CD80^+^CD86^+^ cells in the spleen (*n* = 6). (**F**) Quantification of CD11c^+^CD80^+^CD86^+^ cells in the spleen (*n* = 6). (**G**–**J**) Analysis of serum Th1/Th17-type cytokine levels (*n* = 6). (**G**) TNF-α content in each group, (**H**) IFN-γ content in each group, (**I**) IFN-β content in each group, (**J**) IL-17A content in each group. Data are presented as mean ± SD. A *p*-value < 0.05 was considered statistically significant, with the significance levels marked as * *p* < 0.05, ** *p* < 0.01, *** *p* < 0.001, **** *p* < 0.0001.

To further explore the effects of Neo-BCV combined with CDDP therapy on systemic immunity, we focused on monitoring the spleen weight and spleen index of mice (Figure 2A,B), and determining the immune factors in mouse serum.

The complete maturation of dendritic cells (DCs) and their high expression of co-stimulatory molecules are critical for the effective activation of naive T cells [37,38]. In this study, flow cytometry was used to focus on the activation status of antigen-presenting cells (APCs), especially DCs, in the spleen and tumor tissues. The results showed no significant difference in the proportion of total CD11c^+^ DCs among all groups, indicating that treatment in each group did not alter the total number of splenic DCs (Figure 2C and Figure S1A). However, the proportion of mature DCs (CD11c^+^CD80^+^CD86^+^) expressing high levels of co-stimulatory molecules CD80 and CD86 in the Neo-BCV group and BCV-CDDP group was significantly higher than that in the Control group and the CDDP group (Figure 2D–F). This result indicated that Neo-BCV treatment acts as the core factor driving the functional maturation of DCs. Neo-BCV administration could effectively activate systemic APCs and enhance anti-tumor T cell responses. Notably, there was no significant difference between the Neo-BCV group and the BCV-CDDP group, suggesting that the combined regimen merely retained the immune activation effect of Neo-BCV, and the addition of CDDP failed to further boost systemic anti-tumor immunity.

Serum multiplex cytokine detection results showed that Neo-BCV effectively induced systemic pro-inflammatory responses and Th1-type immune responses, and the addition of Neo-BCV significantly increased TNF-α levels (Figure 2G). Previous studies have demonstrated that IL-17A remodels the tumor microenvironment by regulating immune cells such as macrophages and upregulates PD-L1 expression in tumor cells, thereby promoting the malignant progression of non-small cell lung cancer [39,40]. Results showed that Neo-BCV monotherapy upregulated serum IFN-γ levels, while its combination with CDDP resulted in a reduction in IFN-γ levels. There was no significant difference in IFN-β levels among all groups. Nevertheless, the combined treatment markedly inhibited the expression of IL-17A. (Figure 2H–J). These results indicate that combination therapy modulates systemic cytokine levels and suppresses IL-17A expression, whereas its antitumor effect cannot be fully explained by changes in systemic immune factors alone, suggesting the involvement of other potential mechanisms.

### 3.3. Remodeling Effect of Neo-BCV Combined with CDDP Therapy on the Tumor Immune Microenvironment

Dynamic changes in antigen-presenting cells (APCs) and immune effector cells within the tumor immune microenvironment (TME) directly determine the efficacy of antitumor immune responses [41,42]. Macrophages are highly heterogeneous and are mainly divided into the classically activated M1 phenotype and the alternatively activated M2 phenotype. M1 macrophages mediate antigen presentation and antitumor effects, whereas M2 macrophages contribute to the formation of an immunosuppressive microenvironment and promote tumor immune escape and progression [43,44].

**Figure 3:**
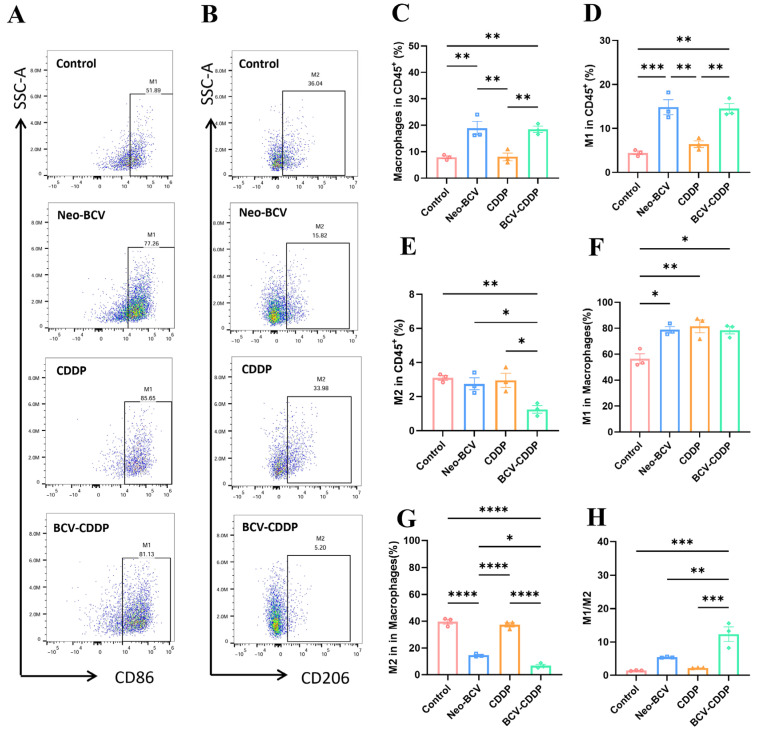
Flow cytometric analysis of M1/M2 phenotypes of macrophages in the tumor microenvironment. (**A**) Proportion of CD86^+^ cells in the TME (*n* = 3). (**B**) Proportion of CD206^+^ cells in the TME (*n* = 3). (**C**) Quantification of CD45^+^ macrophages in the TME (*n* = 3). (**D**) Quantification of CD45^+^ M1 cells in the TME (*n* = 3). (**E**) Quantification of CD45^+^ M2 cells in the TME (*n* = 3). (**F**) Quantification of the frequency of M1 macrophages in total macrophages in the TME (*n* = 3). (**G**) Quantification of the frequency of M2 macrophages in total macrophages in the TME (*n* = 3). (**H**) M1-type Macrophages/M2-type Macrophages ratio in each group (*n* = 3). Data are presented as mean ± SD. A *p*-value < 0.05 was considered statistically significant, with the significance levels marked as * *p* < 0.05, ** *p* < 0.01, *** *p* < 0.001, **** *p* < 0.0001. TME, tumor microenvironment.

To investigate the effects of different treatments on macrophage infiltration and polarization phenotypes in tumor tissues, we performed flow cytometry analysis (Figure 3A,B and Figure S1B). The results showed that the total infiltration proportion of macrophages in tumor tissues of the Neo-BCV group and the BCV-CDDP group was significantly higher than that of the Control group and the CDDP group (Figure 3C), suggesting that Neo-BCV can effectively recruit macrophages to the tumor site. In terms of subset phenotypes, the proportion of M1-type macrophages (CD86^+^) in the Neo-BCV group and the BCV-CDDP group was upregulated, indicating the expansion of APC subsets with strong antigen-presenting capacity in the microenvironment. At the same time, the proportion of M2-type macrophages (CD206^+^) in the BCV-CDDP group was significantly lower than that in other groups, i.e., the BCV-CDDP group had the highest M1/M2 ratio (Figure 3D–H). This indicated that the combination therapy induced M1-type polarization and inhibited M2-type enrichment, ultimately effectively shifting the balance of macrophages to an activated state conducive to anti-tumor effects.

**Figure 4:**
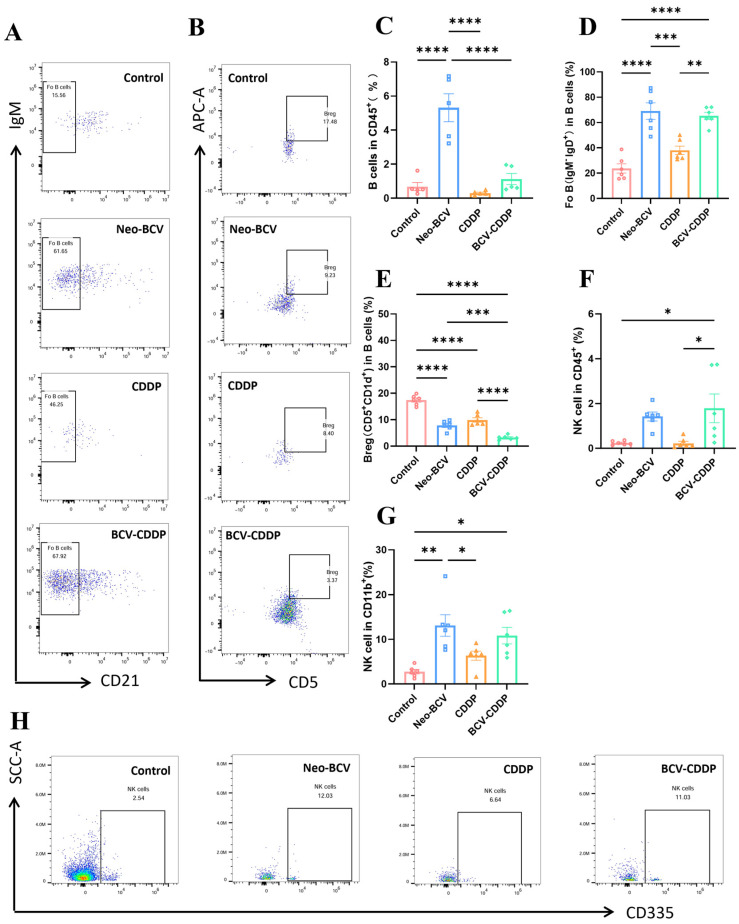
Flow cytometric analysis of B lymphocytes and their subsets as well as NK cells in the tumor microenvironment. (**A**) Proportion of CD21^+^ cells in the TME (*n* = 6). (**B**) Proportion of CD5^+^ cells in the TME (*n* = 6). (**C**) Quantification of B cells in CD45^+^ cells in the TME (*n* = 6). (**D**) Quantification of Fo B cells in the TME (*n* = 6). (**E**) Quantification of Breg cells in the TME (*n* = 6). (**F**) Quantification of CD45^+^ NK cells in the TME (*n* = 6). (**G**) Quantification of CD11b^+^ NK cells in the TME (*n* = 6). (**H**) Proportion of CD335^+^ NK cells in the TME. Data are presented as mean ± SD. A *p*-value < 0.05 was considered statistically significant, with the significance levels marked as * *p* < 0.05, ** *p* < 0.01, *** *p* < 0.001, **** *p* < 0.0001. Fo B cells, follicular B cells; Bregs, regulatory B cells; Tregs, regulatory T cells; NK cells, natural killer cells; TME, tumor microenvironment.

B cells are the core cells in humoral immunity and contain multiple subsets. Among them, follicular B (Fo B) cells mainly mediate T cell-dependent adaptive humoral immunity and can participate in the regulation of immune homeostasis through functional plasticity; another B cell subset, regulatory B cells (Bregs), can inhibit anti-tumor activity by producing immune factors such as interleukin-10 and induce the differentiation of regulatory T cells (Tregs) [45,46,47]. The results showed that compared with CDDP group, Neo-BCV promoted the recruitment of B cells and Fo B, and synergized with CDDP to downregulate the proportion of Bregs (Figure 4A–E and Figure S2A). This positive regulation of the properties of B cell subsets enhances beneficial humoral immunity while further eliminating humoral immunosuppressive components in the microenvironment.

Natural killer (NK) cells are powerful effectors of innate immunity and the first line of defense against cancer, which can eliminate transformed cells while preserving normal healthy cells [48]. The introduction of Neo-BCV increased the infiltration level of NK cells, effectively enhancing the cytotoxicity in the tumor immune microenvironment, with statistically significant differences (Figure 4F–H and Figure S2B).

**Figure 5:**
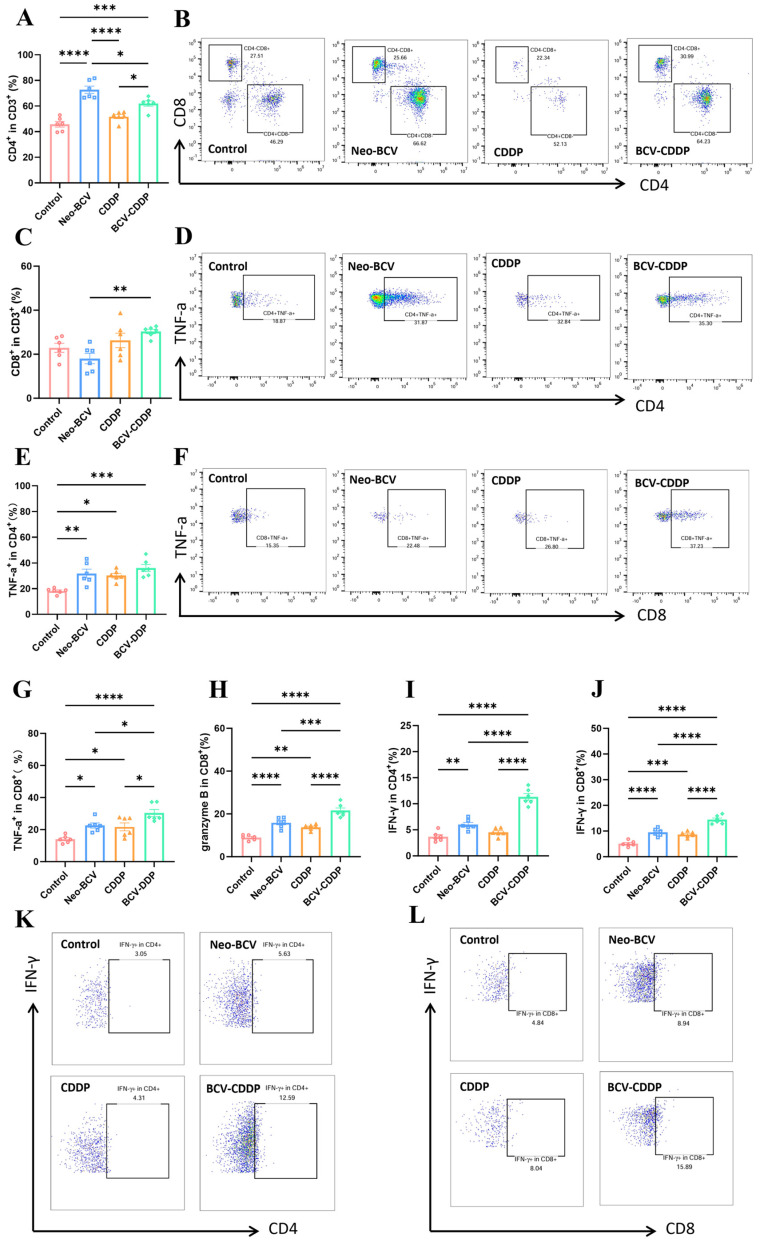
Flow cytometric analysis of T cells, TNF-α-secreting CD4^+^ and CD8^+^ T cells, and analysis of T cell functional activity: IFN-γ and Granzyme B expression in the tumor microenvironment. (**A**) Quantification of CD3^+^ CD4^+^ T cells in the TME (*n* = 6). (**B**) Proportion of CD4^+^ and CD8^+^ cells in the TME (*n* = 6). (**C**) Quantification of CD3^+^CD8^+^ T cells in the TME. (**D**) Proportion of CD4^+^ TNF-α^+^ cells in the TME (n = 6). (**E**) Quantification of CD4^+^ TNF-α^+^ cells in the TME (*n* = 6). (**F**) Proportion of CD8^+^ TNF-α^+^ cells in the TME (*n* = 6). (**G**) Quantification of CD8^+^ TNF-α^+^ cells in the TME (*n* = 6). (**H**) Quantification of CD8^+^ granzyme B^+^ cells in the TME. (**I**) Quantification of CD4^+^ IFN-γ^+^ cells in the TME (*n* = 6). (**J**) Quantification of CD8^+^ IFN-γ^+^ cells in the TME. (**K**) Proportion of CD4^+^ IFN-γ^+^ cells in the TME. (**L**) Proportion of CD8^+^ IFN-γ^+^ cells in the TME. Data are presented as mean ± SD. A *p*-value < 0.05 was considered statistically significant, with the significance levels marked as * *p* < 0.05, ** *p* < 0.01, *** *p* < 0.001, **** *p* < 0.0001. TME, tumor microenvironment.

Analysis of adaptive immune effector cells showed that Neo-BCV combined with CDDP constructed an ideal T cell infiltration profile. Flow cytometry results showed that Neo-BCV significantly promoted the infiltration of helper T cells (CD3^+^CD4^+^ T cells), while CDDP played a leading role in recruiting cytotoxic T cells (CD3^+^CD8^+^ T cells) (Figure 5A–C and Figure S3A). The combination therapy integrated the dual advantages of Neo-BCV in enhancing CD4^+^ helper signals and CDDP in promoting CD8^+^ T cell infiltration, resulting in the highest infiltration level of effector T cell populations at the tumor site.

**Figure 6:**
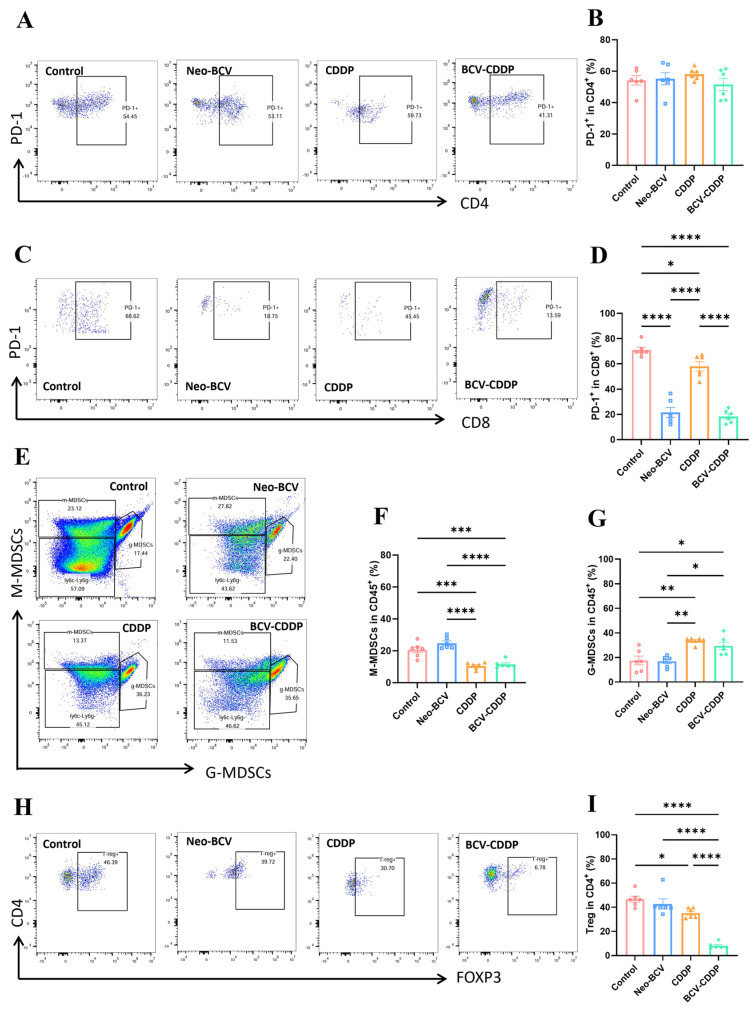
Flow cytometric analysis of PD-1^+^ secreting CD4^+^ and CD8^+^ T cells, MDSCs and Treg cells in the tumor microenvironment. (**A**) Proportion of CD4^+^PD-1^+^ cells in the TME (*n* = 6). (**B**) Quantification of CD4^+^ PD-1^+^ cells in the TME (*n* = 6). (**C**) Proportion of CD8^+^ PD-1^+^ cells in the TME (*n* = 6). (**D**) Quantification of CD8^+^ PD-1^+^ cells in the TME (*n* = 6). (**E**) Proportion of M-MDSCs and G-MDSCs in the TME (*n* = 6). (**F**) Quantification of CD45^+^ M-MDSCs in the TME (*n* = 6). (**G**) Quantification of CD45^+^ G-MDSCs in the TME (*n* = 6). (**H**) Proportion of CD4^+^ Treg^+^ cells in the TME (*n* = 6). (**I**) Quantification of CD4^+^ Treg^+^ cells in the TME (*n* = 6). Data are presented as mean ± SD. A *p*-value < 0.05 was considered statistically significant, with the significance levels marked as * *p* < 0.05, ** *p* < 0.01, *** *p* < 0.001, **** *p* < 0.0001. MDSC, Myeloid-derived suppressor cells; TME, tumor microenvironment.

In addition to changes in cell numbers, we further evaluated the functional activity (cytokine secretion, killer molecule expression) and exhausted state (inhibitory receptor expression) of T cells. The results showed that the tumor-infiltrating CD4^+^ and CD8^+^ T cells in the combination therapy group had a significantly enhanced ability to secrete Th1-type cytokines (TNF-α, IFN-γ), and the level of Granzyme B (a cytotoxic mediator) expressed by CD8^+^ T cells reached a peak, with statistically significant differences (Figure 5D–L and Figure S3A). T cell exhaustion, often marked by high expression of PD-1, is a key cause of impaired function [49,50]. The results showed that although the proportion of PD-1^+^ in CD4^+^ T cells in all treatment groups was not statistically different from that in the Control group, the proportion of PD-1^+^ in CD8^+^ T cells in the Neo-BCV group and the BCV-CDDP group was significantly decreased (Figure 6A–D). This suggested that although not confirmatory as more exhaustion markers are needed, reduced PD-1 expression suggests that exhaustion of T cells is reduced after combination treatment.

In addition, the combination therapy also exhibited attenuation of the function of immunosuppressive cells. Myeloid-derived suppressor cells (MDSCs) are a heterogeneous population of immunosuppressive cells that expand in the tumor microenvironment, infection or inflammatory states. These cells can inhibit anti-tumor immune responses through multiple mechanisms and also interfere with the fine regulation and disordered repair of various cell subsets [51,52].

We further detected the expression levels of two subsets of MDSCs in the tumor microenvironment by flow cytometry (Figure 6E–G and Figure S3B). The experiment found that CDDP was the dominant factor inhibiting the accumulation of monocytic MDSCs (M-MDSCs), and the combination group successfully retained this advantage. For granulocytic MDSCs (G-MDSCs or polymorphonuclear myeloid- derived suppressor cells, PMN-MDSCs), although CDDP monotherapy eliminated M-MDSCs, it induced a compensatory increase in G-MDSCs both in the CDDP and BCV-CDDP treatment groups. Regulatory T cells are another core type of immunosuppressive cells, and the phenomenon that the proportion of regulatory T cells (Tregs) in the combination group decreased to the lowest level (Figure 6H,I) indicated that this treatment regimen can further disrupt the regulatory inhibitory network and achieve in-depth regulation of the immunosuppressive barrier.

### 3.4. Molecular Regulatory Mechanism of Neo-BCV Combined with CDDP Therapy Revealed by Transcriptome Sequencing

**Figure 7:**
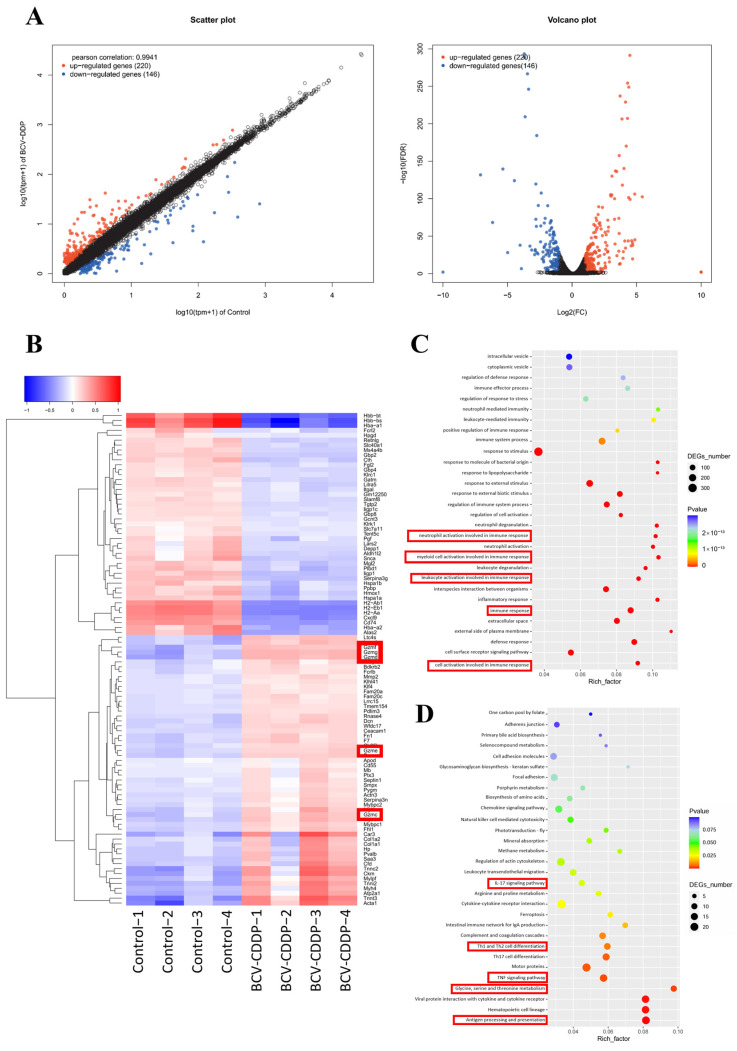
Transcriptome sequencing results and differential analysis. (**A**) Scatter plot and volcano plot of DEG analysis between the BCV-DDP group and the Control group. The horizontal axis represents the fold change in gene expression between the two samples (log2 FC), and the vertical axis represents the significance level of statistical tests (−log10 FDR). Red dots represent significantly upregulated genes, blue dots represent significantly downregulated genes, and black dots represent genes that did not reach the significant threshold. (**B**) Cluster heatmap of DEGs. The color in the figure represents the gene expression level in the samples (*n* = 4), with red indicating high expression and blue indicating low expression. (**C**) GO enrichment analysis bubble chart. The horizontal axis represents the enrichment factor, and the vertical axis represents the enriched GO biological process (BP) terms. The size of the bubble indicates the number of DEGs enriched in the term, and the color indicates the *p*-value. A smaller *p*-value indicates a more significant enrichment result. (**D**) KEGG pathway enrichment analysis bubble chart of DEGs. The horizontal axis represents the enrichment factor, and the vertical axis represents the significantly enriched KEGG pathways. The size of the bubble indicates the number of DEGs enriched in the pathway, and the color indicates the *p*-value. A smaller *p*-value indicates a more significant enrichment result. DEG, differentially expressed genes.

To systematically elucidate the regulatory network of Neo-BCV combined with CDDP in inhibiting tumors at the molecular level, transcriptome sequencing (RNA-seq) was performed on tumor tissues in this study. EdgeR software version R3.6.3 was used to analyze the significance of differential expression of each transcript among samples to identify corresponding differentially expressed genes (DEGs). By comparing the expression levels (TPM values) between the Control group and the BCV-CDDP group, a total of 366 significantly DEGs were identified, including 220 upregulated genes and 146 downregulated genes (Figure 7A).

In addition, cluster analysis was performed on the significantly DEGs and a heatmap was plotted, showing the expression profiles of the top 100 genes with the smallest FDR values. The analysis results showed that compared with the Control group, the BCV-CDDP group exhibited characteristic changes in immune killing pathways. Specifically, the expression levels of key molecules involved in the killing of target cells by cytotoxic T cells and NK cells, such as granzymes (Gzmf, Gzmg, Gzmd, Gzme, Gzmc), were all upregulated (Figure 7B).

At the functional level, GO enrichment analysis results showed that DEGs were significantly enriched in biological processes such as cell activation, cell surface receptor signaling pathway, defense response, immune response and inflammatory response, especially the enrichment of terms related to “activation” and “degranulation” of leukocytes, myeloid cells and neutrophils. This explained the phenomena observed in the previous experiments at the molecular level, including increased infiltration of effector T cells, decreased inhibitory cells and elevated Th1-type cytokines in the tumor microenvironment (Figure 7C). This indicated that the combination therapy constructed a systemic anti-tumor gene expression network by driving a cascade activation from rapid inflammatory response to efficient adaptive immunity.

Further systematic analysis of regulatory hubs by KEGG pathway analysis showed that DEGs were enriched in core immune pathways such as antigen processing and presentation, TNF signaling pathway, Th1/Th2 cell differentiation and IL-17 signaling pathway with statistically significant differences. Among them, the activation of the antigen presentation pathway was consistent with the enhanced function of APCs; the enrichment of cytotoxic pathways confirmed the improvement of T cell function; TNF and IL-17 pathways are not only pro-inflammatory signals but also can directly induce cell death. In addition, changes in a variety of metabolism-related pathways (such as glycine, serine and threonine metabolism) suggested that the combination therapy also remodeled the metabolic state of the tumor microenvironment (Figure 7D). In summary, Neo-BCV combined with CDDP therapy achieved comprehensive remodeling of the tumor immune microenvironment and efficient anti-tumor effects by activating multi-dimensional immune effector pathways and metabolic regulatory networks.

### 3.5. Biosafety Evaluation of Neo-BCV Combined with CDDP Therapy

**Figure 8:**
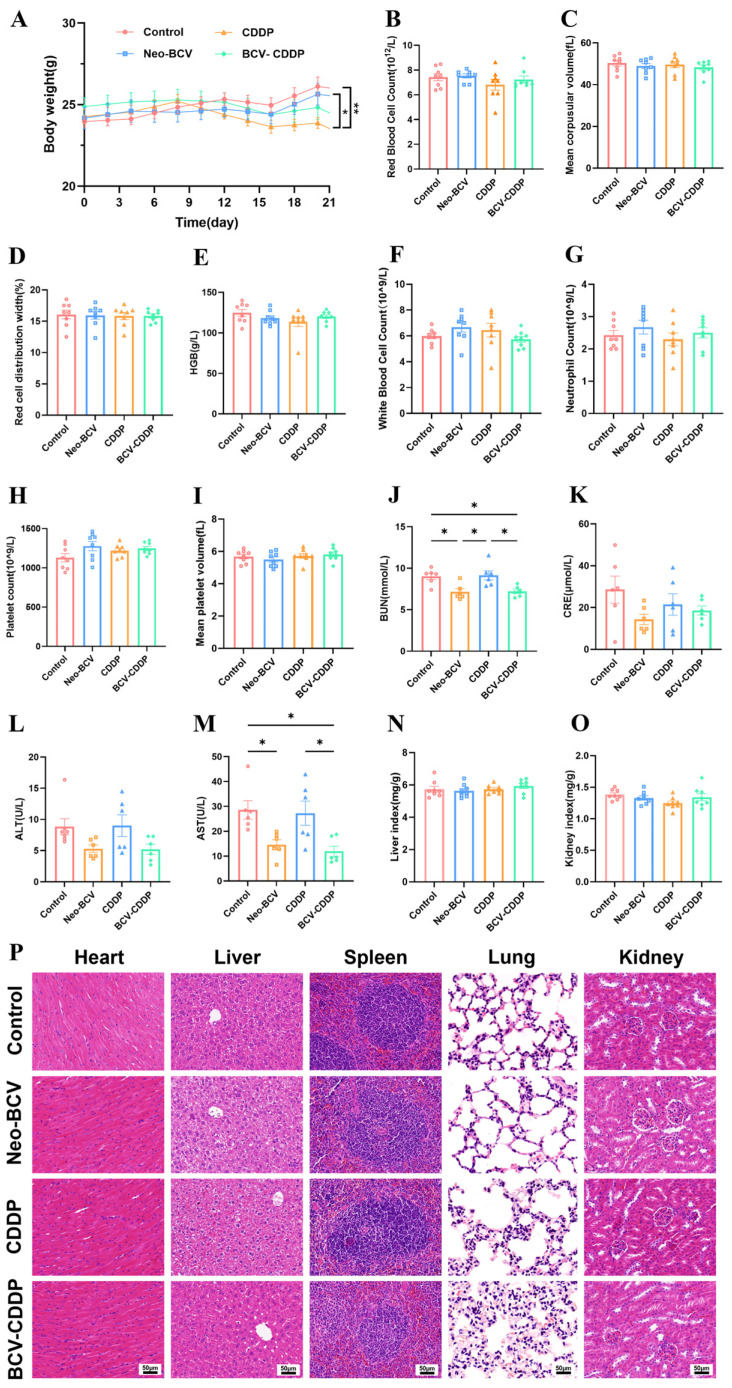
Biosafety evaluation of Neo-BCV combined with CDDP therapy. (**A**) Changes in mouse body weight (*n* = 8). (**B**–**I**) Comparison of main indicators in mouse complete blood count (*n* = 6). (**B**) Red blood cell count in each group. (**C**) Mean corpuscular volume in each group. (**D**) Red blood cell distribution width in each group. (**E**) Hemoglobin content in each group. (**F**) White blood cell count in each group. (**G**) Neutrophil count in each group. (**H**) Platelet count in each group. (**I**) Mean platelet volume in each group. (**J**–**M**) Serum biochemical levels of mice in each group (*n* = 6). (**J**) Renal function indicators (BUN) in each group. (**K**) Renal function indicators (CRE) in each group. (**L**) Liver function indicators (ALT) in each group. (**M**) Liver function indicators (AST) in each group. (**N**) Liver index of mice in each group at the end of the experiment (*n* = 8). (**O**) Kidney index of mice in each group at the end of the experiment (*n* = 8). (**P**) Representative H&E staining images of the heart, liver, spleen, lung and kidney in each group (*n* = 6). Data are presented as mean ± SD. A *p*-value < 0.05 was considered statistically significant, with the significance levels marked as * *p* < 0.05, ** *p* < 0.01.

Besides confirming the synergistic anti-tumor efficacy, this study systematically evaluated the biosafety of Neo-BCV combined with CDDP. In the murine model, CDDP monotherapy caused obvious systemic toxicity with reduced body weight and activity in mice, while the combination therapy effectively alleviated CDDP-induced weight loss and kept mouse body weight within the normal range (Figure 8A). Complete blood count analysis showed that red blood cell, platelet and other key parameters in all groups were normal without statistical differences, indicating the combination therapy caused no significant myelosuppression and had good hematological safety (Figure 8B–I). Serum biochemical tests (Figure 8J–M) revealed abnormally elevated liver and kidney function indicators in the CDDP group, whereas BUN and AST were significantly decreased, while CRE and ALT showed a downward trend without statistical significance, with no statistical differences in liver and kidney indexes among all groups (Figure 8N,O). H&E staining of major visceral organs (Figure 8P) showed no obvious pathological damage in all groups. These findings confirmed that Neo-BCV has significant toxicity-attenuating potential, which ensures the body’s physiological safety while enhancing CDDP’s chemotherapeutic efficacy.

## 4. Discussion

Lung cancer is one of the tumors that seriously threaten human life and health. In recent years, although great progress has been made in lung cancer immunotherapy, the complexity of the tumor immune microenvironment and acquired tumor drug resistance remain the main obstacles limiting clinical efficacy, and researchers are still exploring new and more effective tumor treatment methods [53]. Cancer immunotherapy based on bacterial vaccines has a long history, and in recent years, bacterial therapy has pioneered a new era of cancer immunotherapy [54].

The bacterial composite vaccine Neo-BCV developed by us has been proven in previous studies to promote the maturation and infiltration of dendritic cells at the tumor site, thereby inhibiting tumor growth [33]. In this study, we combined it with CDDP, a classic chemotherapeutic drug, to explore their synergistic anti-tumor potential and clarify the remodeling mechanism of the combination on the tumor microenvironment.

The results of this study showed that the combination group of Neo-BCV and CDDP not only increased the tumor inhibition rate to 87.77% but also significantly alleviated the systemic damage caused by chemotherapy while improving the quality of immune response, achieving the therapeutic goal of “enhanced efficacy and reduced toxicity”. The mechanism of synergistic efficacy enhancement is first reflected in the activation of innate immunity and antigen presentation: Neo-BCV can provide a strong signal for the maturation of dendritic cells, efficiently linking early defense and specific antigen presentation. It also markedly elevates the levels of NK cells, thereby further enhancing the killing efficacy of the body’s innate immunity. The results showed that Neo-BCV significantly upregulated the expression of CD80/CD86 on the surface of splenic DCs, which laid a solid foundation for the subsequent activation of specific T cells. Meanwhile, at the tumor site, Neo-BCV successfully recruited macrophages and induced their polarization from pro-tumor M2 type to anti-tumor M1 type. This transformation of the activation state of APCs not only enhanced the efficiency of antigen processing but also broke the immune tolerance state at the tumor site by secreting Th1-type cytokines such as TNF-α.

The advantage of the combination therapy lies in the in-depth remodeling of adaptive immunity. Experiments confirmed that there is a complementary recruitment mechanism between Neo-BCV and CDDP: the vaccine mainly promotes the infiltration of CD4^+^ T cells to provide helper signals, while CDDP may dominate the recruitment of CD8^+^ T cells by releasing DAMPs. The combination group integrated these dual advantages, resulting in the highest infiltration level of effector T cells and B cells in tumor tissues. More importantly, the combination therapy not only increased the number of cells but also significantly improved their functional quality. The core genes identified by transcriptome sequencing were concentrated in the granzyme family (Gzmf, Gzmg, Gzmd, etc.), which was consistent with the increased secretion of Granzyme B and decreased expression of PD-1 observed by flow cytometry analysis, indicating that the combination therapy enhanced the body’s killing efficiency at both the molecular and cellular levels.

The precise dismantling of the immunosuppressive barrier is one of the key mediators of the combined action of the two drugs. MDSCs are the key mediators of chemotherapy resistance and immunosuppression. The study found that Neo-BCV plays a key role in significantly eliminating M-MDSCs, and combined with the downregulation of the proportions of M2 macrophages, Treg and Breg cells in the combination therapy, it indicated that this regimen can disintegrate the immunosuppressive network in multiple dimensions. This fine regulation of inhibitory cells clears the way for effector T cells to exert their functions.

Owing to its severe toxicities, the clinical application of high-dose CDDP regimens is limited. In this context, Neo-BCV exerts a prominent toxicity-attenuating effect: it can effectively ameliorate CDDP-induced hepatorenal damage, and also reverse the body weight loss observed in mice of the CDDP monotherapy group, which we hypothesize is attributable to the chemotherapy-associated gastrointestinal toxicity induced by CDDP. Our study results demonstrated that the hepatorenal biochemical indicators (BUN, CRE, ALT, AST) in mice of the combination treatment group were reduced. Furthermore, hematoxylin-eosin (H&E) staining results revealed that despite the elevation of systemic inflammatory factors and increased spleen weight, no obvious pathological damage was observed in all major visceral organs of the mice. Taking all the aforementioned evidence into consideration, we reasonably conclude that the systemic inflammation induced by Neo-BCV is characterized as a tightly regulated and tumor-targeted adaptive inflammatory response, which can effectively modulate and maintain systemic immune homeostasis. We hypothesize that Neo-BCV induces the remodeling and reprogramming of systemic immune and metabolic profiles in tumor-bearing mice, thereby suppressing pathological inflammatory responses in normal organs, including the liver, kidney, and gastrointestinal tract. This regulatory effect enables Neo-BCV to exert prominent protective effects, such as alleviating inflammatory damage in hepatic and renal tissues and improving gastrointestinal function, without impinging on the anti-tumor immune response within the tumor microenvironment. However, the specific signaling pathways and underlying regulatory mechanisms responsible for these effects remain to be further elucidated in detail.

Furthermore, the vaccine developed in this study demonstrates high cost-effectiveness, with treatment expenses substantially lower than the current clinical standard—PD-1 inhibitors combined with chemotherapy [55]. Although Neo-BCV is a polyvalent formulation, it poses no risk of infection as all components are thoroughly heat-inactivated. Regarding its safety profile, the immune activation may trigger transient and manageable irAEs—such as the low-grade fever and localized induration observed in our model. However, these acute-phase inflammatory responses are fundamentally distinct from the profound, often chronic autoimmune toxicities and systemic organ damage typically associated with PD-1 blockade [20]. Crucially, this combination therapy effectively preserves organ function and mitigates systemic damage in mice. Consequently, compared to standard PD-1 inhibitors combined with chemotherapy regimens characterized by more severe irAEs, our approach exhibits a superior safety profile.

Despite its contributions, this study has several limitations. First, the subcutaneous LLC model does not fully encapsulate the organ-specific microenvironment of advanced NSCLC; hence, orthotopic or metastatic models are needed for better clinical correlation. Second, the absence of a glucan-only control group limits our ability to precisely isolate the specific immune-activating contributions of the bacterial components. Finally, the molecular mechanisms underlying toxicity alleviation and the efficacy in CDDP-resistant models require further exploration. In addition, tumor tissues from monotherapy groups were not subjected to transcriptome sequencing, and the existing transcriptome data were not further mined for immune cell subset characterization. These limitations will be addressed in future investigations.

## 5. Conclusions

In summary, this study demonstrates that the combination of Neo-BCV and CDDP constructs a robust, multi-dimensional anti-tumor defense network. As a potent immunomodulator, Neo-BCV independently drives the functional maturation of splenic DCs and triggers the systemic elevation of pro-inflammatory cytokines, notably TNF-α. At the tumor site, the vaccine effectively facilitates macrophage recruitment and NK cell infiltration and the accumulation of Fo B cells, thereby strengthening innate immune surveillance.

**Figure 9:**
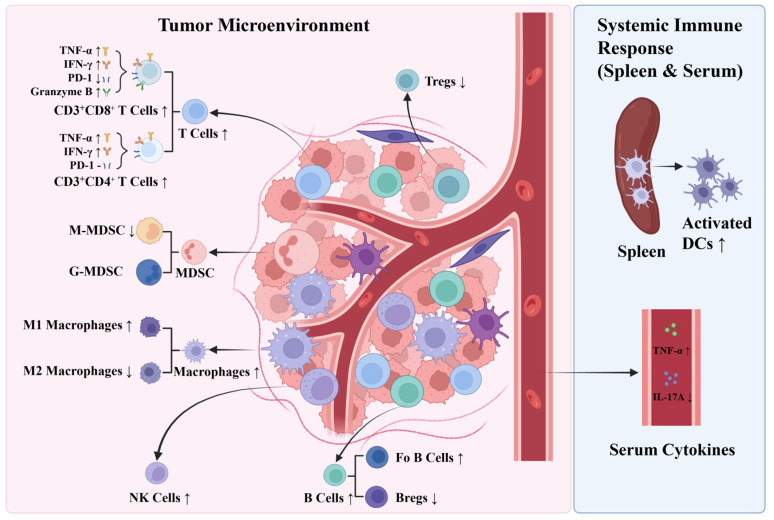
Summary diagram. Neo-BCV combined with CDDP therapy enhances the proportion or function of pro-inflammatory immune cells and suppresses immunosuppressive cells in the tumor microenvironment. At the systemic immune level, this combination increases the number of activated dendritic cells in the spleen, accompanied by elevated pro-inflammatory cytokines and reduced IL-17A in the serum, collectively reflecting an enhanced anti-tumor immune response.

The unique advantage of this combination therapy lies in its ability to synergistically activate APC-mediated antigen presentation and recruit high-efficiency effector T cell populations. This integrated action effectively reverses T cell exhaustion, repairs chemotherapy-induced immune disorders while achieving a profound reduction in immunosuppressive populations including M2 macrophages, M-MDSCs, Tregs, and Bregs. Consequently, this regimen achieved a superior tumor inhibition rate of 87.77% and successfully alleviated CDDP-induced systemic toxicities, including weight loss and hepatorenal damage (Figure 9). These findings provide a solid preclinical foundation for Neo-BCV as a safe and efficient adjunct to conventional chemotherapy in lung cancer treatment.

## Data Availability

The original data presented in this study are included in the article and Supplementary Materials. Further inquiries can be directed to the corresponding authors.

## Supplementary Materials

The following supporting information can be downloaded at: https://www.mdpi.com/article/10.3390/vaccines14050436/s1, Supplementary Figure S1. Flow cytometric gating strategy for Spleen and TME analysis. (A) Flow cytometry gating strategy for DCs in the Spleen. (B) Flow cytometry gating strategy for macrophages in the TME. DCs, dendritic cells; TME, tumor microenvironment; Supplementary Figure S2. Flow cytometric gating strategy for TME analysis. (A) Flow cytometry gating strategy for B cells in the TME. (B) Flow cytometry gating strategy for NK cells in the TME. NK cells, natural killer cells; TME, tumor microenvironment; Supplementary Figure S3. Flow cytometric gating strategy for TME analysis. (A) Flow cytometry gating strategy for T Cells, their subsets and functional molecules in the TME. (B) Flow cytometry gating strategy for MDSCs in the TME. TME, tumor microenvironment; MDSC, Myeloid-derived suppressor cells.
