# Supplementary material for: Enhanced Anti-Lung Cancer Efficacy of Neo-BCV Combined with Cisplatin: Immune Activation and Tumor Microenvironment Remodeling
Source: Vaccines (Basel). 2026 May 13;14(5):436. doi: 10.3390/vaccines14050436 (PMC13211431; doi:10.3390/vaccines14050436)
Supplement: Supplementary file 1 [file vaccines-14-00436-s001.zip › vaccines-4216457-supplementary.pdf]

Supplementary Material

Enhanced Anti-Lung Cancer Efficacy of Neo-BCV Combined with Cisplatin: Immune Activation and Tumor Microenviron-ment Remodeling

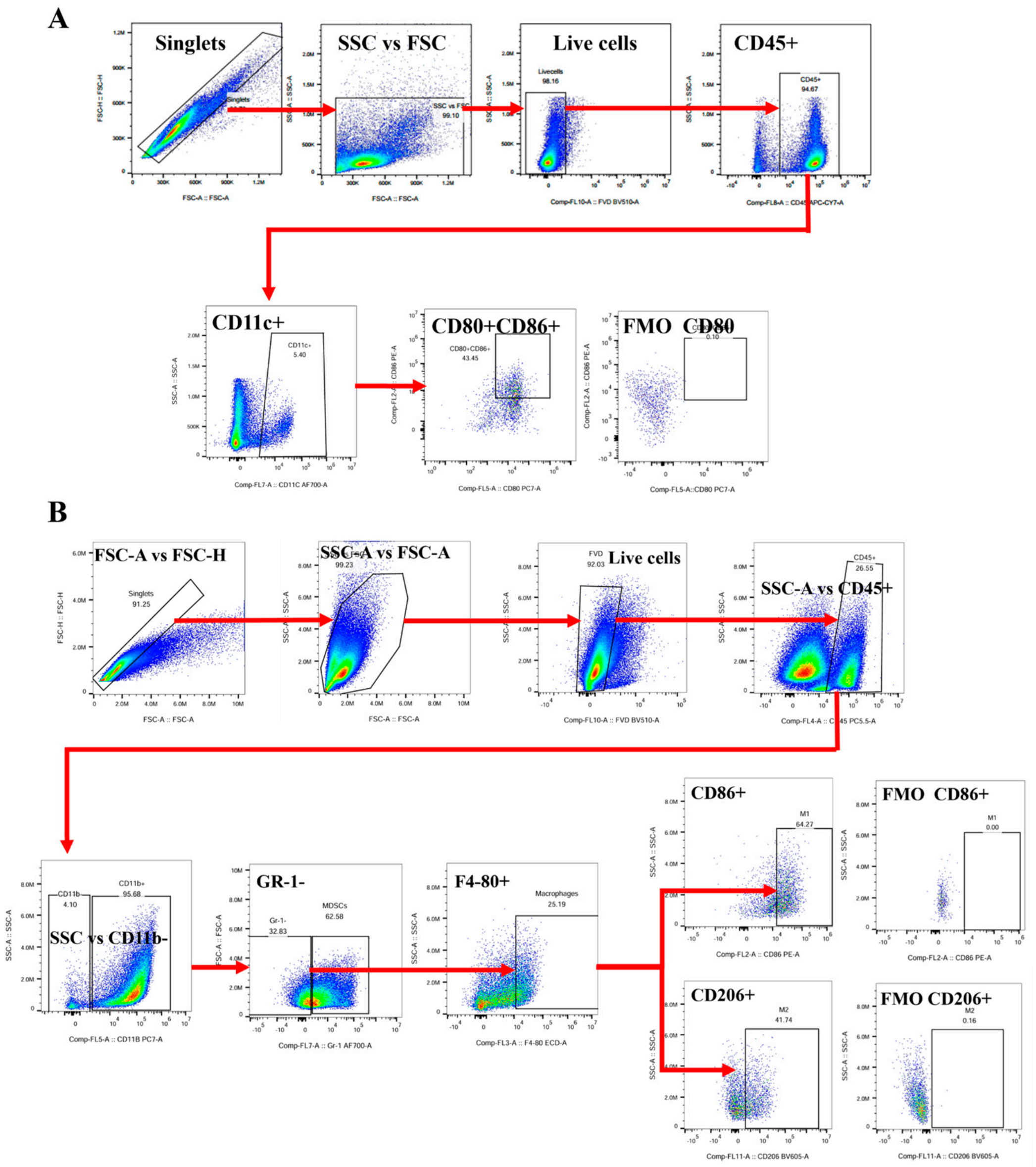

Supplementary Figure S1. Flow cytometric gating strategy for Spleen and TME analysis. (A) Flow cytometry gating strategy for DCs in the Spleen. **(B)** Flow cytometry gating strategy for macrophages in the TME. DCs, dendritic cells; TME, tumor microenvironment.

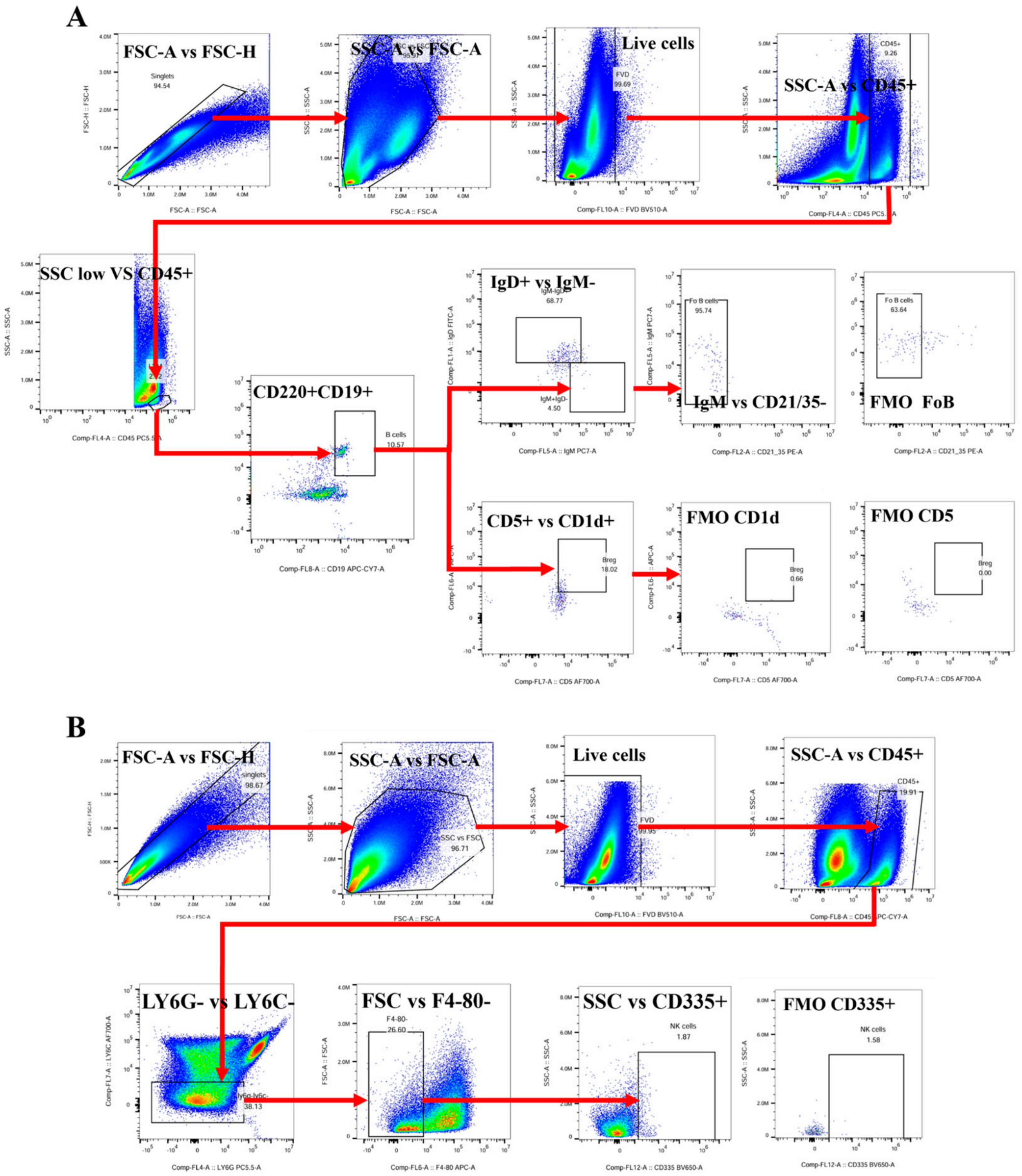

Supplementary Figure S2. Flow cytometric gating strategy for TME analysis. (A) Flow cytometry gating strategy for B cells in the TME. **(B)** Flow cytometry gating strategy for NK cells in the TME. NK cells, natural killer cells; TME, tumor microenvironment.

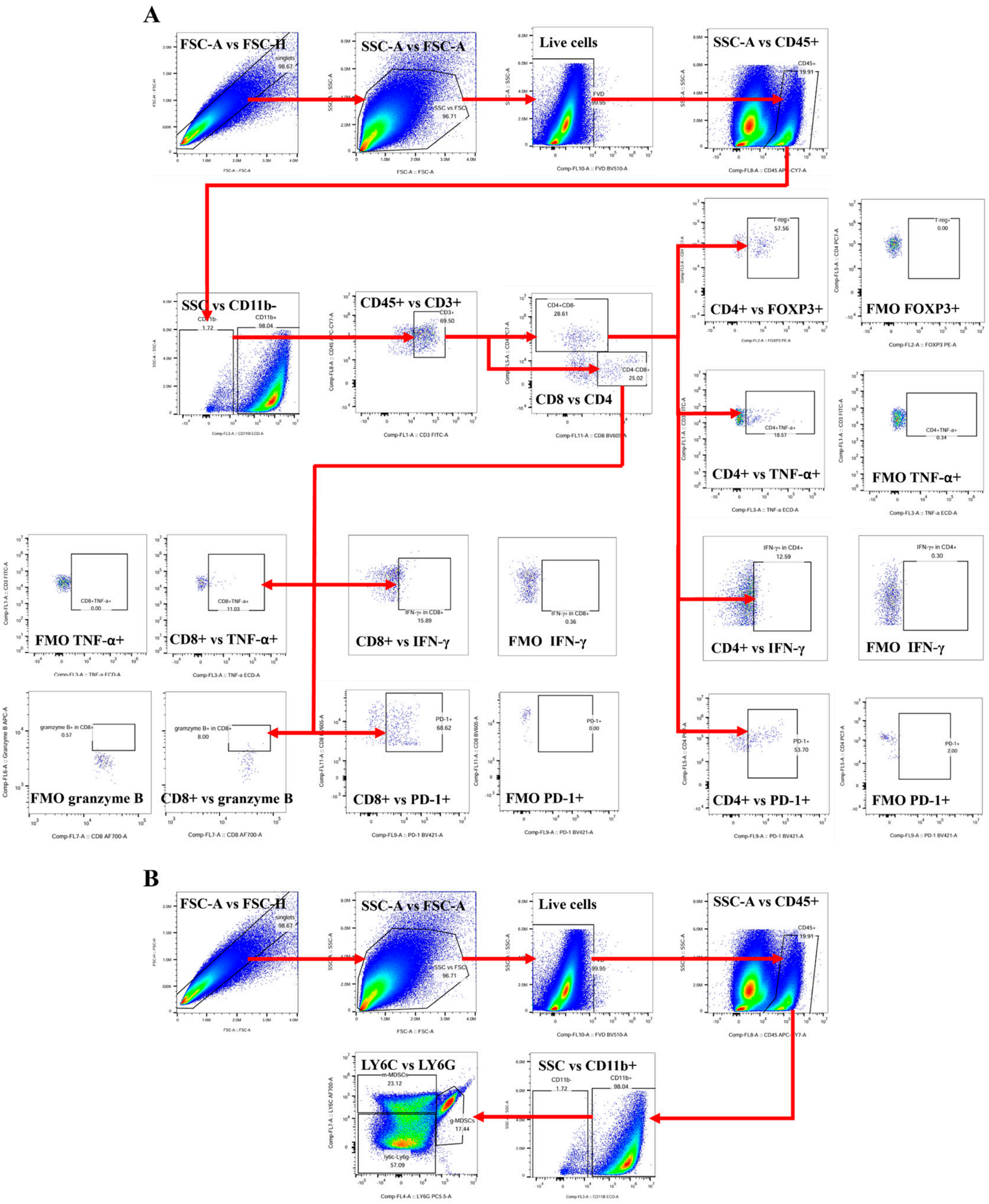

Supplementary Figure S3. Flow cytometric gating strategy for TME analysis. (A) Flow cytometry gating strategy for T Cells, their subsets and functional molecules in the TME. **(B)** Flow cytometry gating strategy for MDSCs in the TME. TME, tumor microenvironment; MDSC, Myeloid-derived suppressor cells.
